# Identification of predictive parameters of juvenile idiopathic arthritis among a cohort of patients with muskoloskeletal pain

**DOI:** 10.1186/1546-0096-12-S1-P194

**Published:** 2014-09-17

**Authors:** Elena Tononcelli, Antonella Meini, Paola Poli, Chiara Gorio, Chiara Mingotti, Marco Cattalini

**Affiliations:** 1 Department of Clinical and Experimental Sciences, University of Brescia, Pediatric Clinic, Pediatric Rheumatology Unit, Brescia, Italy

## Introduction

Musculoskeletal pain is a common complaint in the pediatric population and the children affected are often referred to the rheumatologist. Indeed, although the differential diagnosis in these children may be wide, a carefully guided anamnesis allows to orientate it in the majority of cases.

## Objectives

**T**o recruit children referred to our Centre for musculoskeletal pain and explore if some parameters, obtainable by a thorough medical history evaluation, would be good predictors of the final diagnosis. Secondary aim was to assess the distribution of the different etiologies of muculoskeletal pain in a cohort of patients referred to the pediatric rheumatologist.

## Methods

Clinical records of children referred to our Unit for musculoskeletal complaints between June 2011 and December 2013 were collected during the first evaluation. A database was built, considering: characteristics of pain (frequency, pattern, precipitating factors), joint swelling, morning stiffness, constitutional symptoms, laboratory test results (ESR, CRP, ANA, RF), family history. After the final diagnosis was made, each patient was categorized according to three subgroups: JIA, post-infective arthritis, non-inflammatory disorders and the three categories were compared for each one of the parameters recorded. Leave-on-out cross validation and logistic regression model were used to identify the parameters associated with the final diagnosis of JIA.

## Results

A total of 178 children were evaluated throughout this study. The final diagnosis were: JIA (36), infection-related arthritis (28), non-inflammatory disorders (114). The comparison between the three groups showed a pattern of signs and symptoms specific for each one of the categories, in particular JIA and non-inflammatory disorders: persistent joint swelling, the rest as precipitating factor, the presence of morning stiffness and a persistent pain were statistically associated with the diagnosis of JIA (all p values<0.0001), while absence of joint swelling, activity as a precipitating factor, recurrent pain on evenings/nights, resulted statistically associated with non-inflammatory disorders (all p values<0.001). Focusing on JIA diagnosis, we then analyzed the impact of each one of the identified parameters on the final diagnosis, obtaining the formula y = k + b1x1 + b2x2 + b3x3 + b4x4 (Y= probability of having JIA; k=15,735; x1= joint swelling’s pattern; x2= precipitating factors; x3=morning stiffness; x4=pain frequency). This formula gives the probability that a child with musculoskeletal complaints will receive a final diagnosis of JIA (sensitivity 90.9%, specificity 95.3%).

## Conclusion

Musculoskeletal pain is a common cause of rheumatological referral, but only a minority of patients will receive a diagnosis of JIA, indeed the majority of cases are secondary to non- inflammatory disorders. A detailed evaluation of the medical history turned out to be a valid tool to drive the differential diagnosis with high sensitivity. The obtained formula could be used to calculate the probability that a patient with musculoskeletal complaints could be affected by JIA. Obviously this formula cannot be interpreted as a substitute of a specialized evaluation but, if our results will be confirmed on larger cohort, it may be a valuable tool for primary care physician to address the differential diagnosis and the diagnostic work/up.

## Disclosure of interest

None declared.

